# Changes in functional connectivity are associated with functional independence in the early postoperative period following awake surgical resection of language-eloquent glioma

**DOI:** 10.1093/noajnl/vdaf192

**Published:** 2025-09-02

**Authors:** Kyle R Noll, Evan D Bander, Henry S Chen, Mariana Bradshaw, Jeffrey S Wefel, Vinodh A Kumar, Sujit S Prabhu, Ho-Ling Liu

**Affiliations:** Department of Neuro-Oncology, The University of Texas MD Anderson Cancer Center, Houston, TX, USA; Department of Neurosurgery, The University of Texas MD Anderson Cancer Center, Houston, TX, USA; Department of Radiology, University of Colorado School of Medicine-Anschutz, Aurora, CO, USA; Department of Neuro-Oncology, The University of Texas MD Anderson Cancer Center, Houston, TX, USA; Department of Radiation Oncology, The University of Texas MD Anderson Cancer Center, Houston, TX, USA; Department of Neuro-Oncology, The University of Texas MD Anderson Cancer Center, Houston, TX, USA; Department of Neuroradiology, The University of Texas MD Anderson Cancer Center, Houston, TX, USA; Department of Neurosurgery, The University of Texas MD Anderson Cancer Center, Houston, TX, USA; Department of Imaging Physics, The University of Texas MD Anderson Cancer Center, Houston, TX, USA

**Keywords:** activities of daily living, brain tumor, connectomics, functional independence, glioma

## Abstract

**Background:**

Neurocognitive decline in patients with primary brain tumors is associated with alterations in the functional connectome and reduced independence in daily living. This study explores postoperative connectomic changes associated with functional independence outcomes in patients with eloquent glioma, and how these associations differ from neurocognitive-connetcomic relationships.

**Methods:**

Fifteen patients with left perisylvian glioma underwent resting-state functional magnetic resonance imaging (fMRI) and neuropsychological evaluation within 2 weeks before and on average 1 month after resection. Functional independence was measured with the Physical Self-Maintenance Scale (PSMS) and the Instrumental Activities of Daily Living scale (IADL). Graph theoretical analysis quantified functional brain network properties.

**Results:**

Postoperative need for assistance in at least 1 activity on the IADL increased in 80% of patients with Total scores significantly increasing at the group level (Mdn change = 4.0, *P* = .006). In contrast, need for assistance on the PSMS increased in less than 30% of patients and Total scores were unchanged. Connectomic changes in Local Efficiency, Clustering Coefficient, Path Length, and Betweenness Centrality showed significant associations with need for assistance on the IADL (ρ = 0.63 to.72, all *P* < .01) but few activities on the PSMS. Functional independence ratings were not associated with Karnofsky Performance Status, manual dexterity, tumor volume, or extent of resection.

**Conclusions:**

Alterations in functional connectomic properties after eloquent glioma resection are associated with early postoperative need for assistance in instrumental activities. Changes in connectomics are also associated with cognitive outcome in this population, though properties most involved appear to differ from those underlying changes in independence.

**Key Points**

- Glioma resection induces changes in patient functional connectomes.
- Connectomic alterations are associated with neurocognitive and daily independence outcomes.
- Network integration may be more critical for daily activities than specific cognitive abilities.

**Importance of the Study**

- This preliminary work adds to the very limited existing research regarding associations between functional connectomics and patient outcomes following glioma resection, representing the first to employ resting-state fMRI and dedicated measures of activities of daily living both prior to and following tumor resection. Importantly, postoperative connectomic alterations appear strongly related to the ability to independently manage critical daily activities. In contrast, routinely available clinical data bear little relationship to patient functional outcomes. These findings represent a first step toward future larger-scale work developing connectomics-based outcome prediction models with consideration of important patient and tumoral features. With validation, such models may ultimately lead to enhanced surgical planning techniques and mitigation of surgically induced connectomic changes and associated functional decline.

Primary brain tumors, such as gliomas, and their treatment can have profound impact upon patient neurocognitive and functional status.^1,2^ Surgical resection of gliomas must balance the onco-functional goals of obtaining a maximal resection to improve survival and reduce oncological burden, while striving to minimize risk of postoperative decline in neurocognitive and/or motor function.^3–7^ Patients with tumors near language-eloquent structures in the dominant hemisphere may harbor particular risk of neurocognitive decline.^8^ Techniques such as awake craniotomy, functional brain mapping, neuro-navigation, and tractography can help reduce risk of iatrogenic deficits in these high-risk patients.^9–14^ However, some studies estimate 40% to 60% exhibit postoperative neurocognitive decline despite use of such operative adjuncts, most notably in aspects of memory and executive functioning.^8,15–18^ Unfortunately, early postoperative deficits in higher-order domains may persist in a substantial proportion of patients with eloquent glioma,^18^ in contrast to more basic language skills that often recover over time.^19^

Variability in outcomes may relate in part to the limited utility of language mapping for prediction of outcomes across diverse neurocognitive functions. Pre- and intraoperative language mapping is particularly useful for identifying discrete brain regions involved in basic speech and language abilities. Sparing critical areas from surgical damage reduces the likelihood of patients with eloquent glioma developing persistent surgically acquired aphasia.^8,9,13^ However, identifying and sparing essential brain regions underlying higher-order neurocognitive abilities (including some complex linguistic functions) is challenging with conventional localizationist mapping approaches.^20^ Evidence indicates that many complex neurocognitive abilities depend upon the integrity of widespread and distributed functional networks.^21^ Fortunately, advances in resting-state functional magnetic resonance imaging (fMRI) include methods to reliably measure the structure, properties, and behavior of whole-brain networks, or the connectome. Connectomic approaches offer a more complete picture of brain networks underlying neurocognition, including multifaceted functions (eg, executive functioning) often challenging to comprehensively interrogate with task-based techniques.^22^

By moving beyond traditional localizationist methods, connectomics-informed surgical planning has potential to improve patient outcome prediction and preservation of quality of life. However, better understanding of relationships between surgically induced perturbations of the connectome and postoperative patient outcomes is needed before clinical application can be seriously considered. Some early work is encouraging, indicating that pre- to postoperative changes in connectomic properties are associated with neurocognitive outcomes following brain tumor resection.^16,23,24^ In language-eloquent glioma more specifically, evidence suggests that postoperative worsening in language, executive functioning, and learning and memory are particularly related to alterations in connectomic properties characterizing nodal similarity and centrality (“hubness”).^16^ Importantly, a number of intrinsic networks appear lateralized, including the typically left-lateralizing default mode network important to varied neurocognitive abilities.^25^ This may explain some of the greater-risk of neurocognitive decline in patients following dominant (ie, usually left) hemisphere glioma resection.

Neurocognitive impairment is associated with reduced functional status and patient ability to independently perform basic and instrumental activities of daily living, with memory and executive deficits appearing most predictive of increased need for assistance.^2,6^ While assessment of functional independence is increasingly recognized as an important outcome in glioma research and practice,^6,7,9^ documented rates of decline in daily functions vary widely across studies. Additionally, few studies utilize dedicated measures quantifying abilities in both basic (eg, self-care) and higher-order instrumental activities (eg, medication and financial management) necessary for sustaining independence and autonomy in life.^6^ More often, these functional outcomes are extracted from patient-reported measures of health-related quality of life, which can be biased by variability in patient insight (eg, anosagnosia) or affective distress.^26^ Caregiver ratings can reduce such bias, especially when rating need for assistance in daily activities given the caregiver’s direct role in these aspects of patient care. Unfortunately, there remains a paucity of work investigating patient functional independence using validated informant-ratings of activities of daily living, particularly in those with eloquent glioma who harbor greatest risk of neurocognitive decline. It is also unclear how relationships with surgically-induced alterations in the functional connectome may differ across neurocognitive and functional independence outcomes, despite known dependence of patient autonomy upon neurocognitive capacity.^2^

This study explores patient functional independence outcomes following resection of eloquent glioma, and relationships with neurocognitive and whole-brain functional changes. It is hypothesized that functional connectomic changes will relate to both neurocognitive and functional independence outcomes following tumor resection. Intuitively, these behavioral outcomes may be expected to share similar underlying connectomic alterations, though potential differences require exploration.

## Materials and Methods

Adult right-handed patients with newly identified suspected glioma were prospectively recruited at The University of Texas MD Anderson Cancer Center from 2017 to 2021. Potential cases were identified by the imaging team upon receipt of orders for preoperative neuroimaging studies. Cases were considered for inclusion if patients (1) had unifocal lesions near left hemisphere language-eloquent cortex (per Sawaya et al.^27^); (2) had lesions identified as glioma via pathology report or suspected to represent glioma per radiologist interpretation of preoperative neuroimaging; (3) were native English speakers; (4) were right-handed; (5) were able to undergo magnetic resonance imaging (MRI) at 3T; and (6) had planned awake craniotomy for resection of the lesion. Accordingly, patients meeting these criteria reflect typical clinical cases presenting with newly diagnosed language-eloquent tumors that receive preoperative brain mapping and undergo awake craniotomy for standard of care treatment. Those with history of other neurological diseases were excluded, with the exception of seizure disorder secondary to tumor. Patients with a history of prior open surgical resection, radiotherapy, or any type of chemotherapy were also excluded. Twenty-three patients met criteria for the study and were enrolled. Of these, 6 were lost to follow-up MRI or neuropsychological assessment, 1 encountered scanner malfunction, and 1 was unable to tolerate postoperative MRI, leaving a sample of 15 patients for analyses. Participation was voluntary and patients were not compensated. All study procedures were performed as part of standard of care, with the exception of postoperative functional neuroimaging which was performed for research purposes only. The University of Texas MD Anderson Cancer Center Institutional Review Board approved the study and all patients provided written informed consent for participation and collection of standard of care and research imaging and clinical data.

### Functional Independence Assessment

#### Activities of daily living

All patients underwent neuropsychological evaluations within 2 weeks prior to awake craniotomy for tumor resection and again within an average of 1 month after resection but prior to initiation of radiation and/or chemotherapy. As patients were seen for standard of care neuropsychological evaluations, timing of postoperative testing coincided with postoperative neurosurgical follow-up visits, which occur within this timeframe at our institution. This interval also allows more specific interrogation of effects related to surgery without concern for confounding from initiation of radiation and/or chemotherapy. Functional independence assessments consisted of the Physical Self-Maintenance Scale (PSMS) and Instrumental Activities of Daily Living scale (IADL), both of which were completed by informant-caregivers as part of the preoperative and postoperative neuropsychological evaluations.^28^ Both measures are well-validated and have acceptable psychometric properties, including test-retest reliability (Intraclass Correlation Coefficients = 0.59 to.93).^28–30^ They have also been utilized as measures of functional independence in neurologic populations,^30^ including brain tumors.^31^ The PSMS consists of 8 items assessing basic activities of daily living (eg, dressing, bathing, etc.) with each reflecting a different basic task rated on a scale from 1 (total independence) to 5 (total assist). Total scores range from 6 to 30 with greater scores indicating more overall need for assistance in basic activities.

The IADL consists of 8 items assessing more complex instrumental activities of daily living with each item reflecting a different instrumental task (eg, phone use, shopping, medication management, etc.). Possible item ratings range from 1 (total independence) to either 3, 4, or 5 depending on the specific item (maximal scores represent total assist). Total scores range from 8 to 31, and again, greater scores indicate less overall functional independence with instrumental activities. See Table 1 for specific activities assessed by the PSMS and IADL and ranges for item ratings. For both measures, an item rating greater than 1 indicates at least some need for assistance in that activity. Pre- to postoperative change (ie, difference score) on PSMS and IADL items of at least 1 represent either decrease (+ change) or improvement (- change) in functional independence. For instance, a rating of 2 on the IDAL “Shopping” item indicates that the patient is able to independently shop for small purchases, while a rating of 3 indicates the patient needs to be accompanied by a caregiver on any shopping trip. Zero pre- to postoperative change indicates stability in level of independence in the activity.

**Table 1.** Measures of activities of daily living and item content

PSMS^a^	IADL
Toileting	Phone Use^b^
Feeding	Shopping^b^
Dressing	Food Preparation^b^
Grooming	Housekeeping^a^
Ambulation	Laundry^c^
Bathing	Transportation^a^
	Medication Management^c^
	Financial Management^c^

Abbreviations: PSMS, Physical Self-Maintenance Scale; IADL, Instrumental Activities of Daily Living scale.

Item rating ranges: ^a^1 – 5, ^b^1 – 4, ^c^1 – 3; higher ratings reflect greater need for assistance.

#### Performance status

Karnofsky Performance Status (KPS) ratings were extracted from chart review of clinical exams most proximal (within 1 week) to the pre- and postoperative neuropsychological evaluations. KPS represents a physician rating of functional status on an ordinal scale from 0 to 100 in 10-point increments, with 100 indicating complete functional independence and 0 indicating death. Per prior literature,^32^ pre- to postoperative KPS change scores of 20 were described as clinically meaningful, though given the considerable change in function across ratings (eg, 80 to 70), rates of change of at least 10 are also reported and utilized for analyses.

### Motor and Neurocognitive Function Assessment

Patients also completed the Lafayette Grooved Pegboard pre- and postoperatively as part of neuropsychological assessment. The Grooved Pegboard test is a well-validated, objective, performance-based measure of speeded upper extremity fine motor dexterity,^33^ and has been incorporated in prior investigations in glioma.^34^ Completion times are recorded for the dominant and non-dominant hands and converted to demographically-adjusted z-scores (M = 0.0, SD = 1.0) from published normative data stratified by age, education, gender, handedness, and ethnicity.^35^ Reliable change index (RCI) methodology was utilized to determine significant pre- to postoperative change in dexterity at the individual level, accounting for test-retest reliability, measurement error, and practice effects.^36^ While the central focus of this study pertains to functional independence outcomes, our prior work reports neurocognitive data from this sample.^16^ For convenience and to aid comparison with the present results, neurocognitive test results (demographically adjust z-scores; RCI change rates), are presented along with the functional independence data where relevant. Neuropsychological tests administered and abbreviations are indicated in Table 3. Neuro-oncology or neurosurgery clinic notes in closest proximity to assessments (within 1 week) were also reviewed to capture physician-reported pre- to postoperative change (worsened vs. not) in gross strength/hemiparesis from the pre- to postoperative period.

**Table 3.** Measures of pre- and postoperative independence, motor and neurocognitive function, and connectomic properties

	Preoperative	Postoperative		
*N* = 15	Mdn (IQR)	Mdn (IQR)	*P* ^†^	Effect Size^††^
**Functional Independence**				
PSMS Total	6.00 (0.0)	6.00 (1.0)	.246	.30
IADL Total	9.00 (3.0)	15.00 (7.0)	**.006**	.72
KPS^a^	90.00 (20.0)	90.00 (10.0)	.589	.14
**Manual Dexterity (z-score)** Grooved Pegboard^b^				
Right hand	-0.43 (1.19)	-0.83 (1.02)	.197	.33
Left hand	-0.70 (1.02)	-0.73 (0.75)	.437	.20
**Neurocognitive Function (z-score)** *Attention*				
WAIS-IV Digit Span	-0.33 (1.33)	-0.33 (2.00)^a^	.178	.35
WAIS-IV Arithmetic	-0.33 (1.34)	-0.50 (2.00)	.322	.26
*Learning and Memory*				
HVLT-R Total Recall	-1.79 (1.79)	-2.41 (2.89)	.053	.50
HVLT-R Delayed Recall	-1.94 (3.60)	-2.88 (4.09)	.052	.50
HVLT-R Recognition	-1.00 (1.82)	-2.71 (2.73)	**.035**	.54
*Processing Speed*				
WAIS-IV Coding	-0.33 (1.66)	-0.67 (2.33)	.065	.48
WAIS-IV Symbol Search	-0.33 (2.67)^a^	-1.00 (2.33)^a^	.171	.37
TMT Part A	0.39 (1.15)	-0.30 (2.22)	**.020**	.60
*Executive Function*				
TMT Part B	-0.20 (1.68)	-1.10 (4.95)	**.020**	.60
WAIS-IV Similarities	0.00 (0.67)	-0.67 (1.83)^b^	**.013**	.69
MAE COWA	-1.14 (1.75)	-2.10 (2.26)	**.013**	.64
Animals	-1.30 (2.30)	-1.85 (2.73)^a^	.181	.36
*Language*				
MAE Token Test	0.44 (1.05)	-0.44 (2.15)^a^	**.008**	.71
Boston Naming Test	-1.60 (1.10)	-1.30 (2.50)	.699	.10
*Visuospatial Function*				
WAIS-IV Block Design	0.00 (1.33)	-0.17 (1.42)	.645	.12
**Connectomic Properties** *Integration*				
Global Efficiency^c^	0.531 (0.023)	0.533 (0.026)	.733	.09
Characteristic Path Length^c^	2.192 (0.122)	2.169 (0.094)	.233	.31
*Segregation*				
Modularity	0.338 (0.070)	0.342 (0.093)	.955	.01
Clustering Coefficient	0.576 (0.037)	0.576 (0.050)	.691	.10
Local Efficiency	0.758 (0.018)	0.745 (0.049)	.496	.18
*Centrality*				
Betweenness Centrality	104.444 (9.667)	99.800 (12.133)	**.031**	.56
*Resilience*				
Assortativity	0.293 (0.132)	0.210 (0.131)	**.027**	.57

Abbreviations: PSMS, Physical Self-Maintenance Scale; IADL, Instrumental Activities of Daily Living scale; KPS, Karnofsky Performance Status scale; WAIS-IV, Wechsler Adult Intelligence Scale-Fourth Edition; HVLT-R, Hopkins Verbal Learning Test-Revised; TMT, Trail Making Test; MAE, Multilingual Aphasia Examination; COWA, Controlled Oral Word Association.

^a^ *N* = 14; ^b^*N* = 13; ^c^Excluding infinite path lengths.

^†^Pre- and postoperative measures compared with matched pairs Wilcoxon signed-rank tests.

^††^Effect size (r) calculated as the absolute (positive) standardized test statistic (Z) divided by the square root of the number of pairs.

Bolded; significant difference, *P* ≤ .05.

### Neuroimaging

Pre- and immediate (24 to 48 h) postoperative tumor volumetrics from T1-weighted with and without contrast and T2 fluid attenuated inversion recovery (FLAIR) MRI were extracted from the neurosurgery clinical database. Extent of resection was calculated from both FLAIR and T1-weighted MRI. Given inclusion of enhancing and non-enhancing tumors, the greatest of post-contrast enhancing T1 hyperintense or hypointense volumes were used for volumetrics and extent of resection calculations. To characterize potential influence of perioperative infarct upon outcomes, radiologist interpretation of immediate postoperative diffusion weighted imaging (DWI) was reviewed for presence of restricted diffusion. Each patient also underwent fMRI of the brain within 10 days before neurosurgery and again about a month following tumor resection but prior to initiation of radiation or chemotherapy. Preoperative functional neuroimaging was performed as part of standard of care, postoperative functional imaging was performed for research purposes only, and both protocols were consistent with expert consensus recommendations for resting-state fMRI in brain tumor populations.^37^ All scans were performed on the same 3T scanner with full brain coverage. T1 anatomical (IR-prepared 3D FSPGR, TR/TE/TI = 6.1/2.1/400 ms, FA = 20°, 1.0 × 1.0 × 1.2 mm^3^ voxel) and T2 FLAIR (TR/TE/TI = 10000/142/2250 ms, 1.0 × 1.0 × 2.0 mm^3^ voxel) scans were acquired, in addition to resting-state fMRI via a gradient-echo T2*-weighted echo-planar imaging (EPI, TR/TE = 2000/25 ms, 3.8 × 3.8 × 4.0 mm3 voxel, 180 volumes) sequence. For rs-fMRI, patients were instructed to relax, remain awake, and think of nothing in particular for the duration of the 6-min scan. This was the first sequence after the localization scans in each session to prevent patients from falling asleep.

Resting-state fMRI was processed at the individual patient level in MATLAB (Mathworks, Natick, MA) via Statistical Parametric Mapping (SPM 12, Wellcome Department of Cognitive Neurology, London) with the Brain Connectivity Toolbox^38^ and Group ICA of fMRI Toolbox (GIFT).^39^ The resting-state fMRI preprocessing included slice timing correction, realignment, motion correction, regressing out covariates including six motion curves and time curves from white matter and cerebrospinal fluid, spatial smoothing (4 mm FWHM), and bandpass filtering (0.01-0.1 Hz). Data were segmented into 90 cortical and subcortical regions of interest (ROIs) from the Automated Anatomical Labeling (AAL) atlas^40^ after normalization to the Montreal Neurological Institute (MNI) space using the DARTEL toolbox^41^ in SPM 12. Correlations between time curves averaged over each ROI were calculated, and Fisher Z transformation was applied to construct the functional connectivity matrices for each patient, which were then thresholded to a connectivity density of 0.2 and binarized. This threshold is commonly utilized in the connectomics literature and has been found to be among the best densities for preserving meaningful connectivity while mitigating noisy or spurious edges.^42^ This also represents the threshold utilized in our prior work,^16^ facilitating consistency for comparisons of results across studies. From the individual connectivity matrices, networks were constructed via application of graph theory yielding unweighted graphs with nodes representing brain regions and edges constituting the functional relationships between them. Graph theoretical connectomic properties were calculated at the whole-brain level for both pre- and postoperative graphs of each patient using the Brain Connectivity Toolbox,^39^ including measures of integration (Global Efficiency, Characteristic Path Length), segregation (Modularity, Clustering Coefficient, Local Efficiency), centrality (Betweenness Centrality), and resilience (Assortativity).^43^

Global efficiency is the average inverse shortest path length, representing efficiency of information transfer across the network. Characteristic path length is another measure of functional integration and is the average shortest path length between all pairs of nodes. Modularity measures segregation, quantifying the degree to which the network can be subdivided into non-overlapping functional groups. It is the proportion of all edges connecting nodes in a given module minus the proportion of expected edges if distributed at random. Clustering coefficient measures how strongly a network is locally connected and consists of the number of edges between direct neighbors of a node proportionate to the maximum number of possible connections between those nodes. Local efficiency is another measure of segregation and represents the global efficiency computed on node neighborhoods, and as such, is related to the clustering coefficient. As the name implies, betweenness centrality measures centrality or the degree to which brain hubs interact with other regions. It is calculated as the fraction of all shortest paths in the network passing through a given node, averaged across the brain. Finally, assortativity is a measure of network resilience or robustness and is the correlation between the degrees of all nodes on two opposite ends of an edge. Network measures exhibit good to excellent longitudinal reproducability.^44^ For more detailed descriptions of network connectivity measures and their mathematical representations, see Rubinov and Sporns.^43^

### Statistical Analysis

Group level comparisons of pre- and postoperative Total PSMS and IADL scores, KPS scores, Grooved Pegboard T-scores, and connectomic properties were conducted with matched pairs Wilcoxon signed-rank tests. Change scores were also examined, calculated as the difference between postoperative and preoperative connectomic, functional independence, KPS, motor, and neurocognitive measures for each patient. To determine whether changes in independence measures were potentially influenced by motor difficulties (rather than mental capacity), correlations between pre- to postoperative PSMS and IADL change scores, Grooved Pegboard T-score change, and motor decline on neurologic exam (yes/no) were examined with Spearman rank-order correlations (ρ) and point biserial correlations (rpb). Correlations between pre- to postoperative change in connectomic network measures and changes in independence measures were determined with Spearman rank-order correlations (ρ). Additionally, associations between pre- to postoperative changes in functional independence and neurocognitive function were also explored with Spearman correlations (ρ) to better understand relationships between functional independence and neurocognitive outcomes. As recommended by Cohen,^45^ Wilcoxon test effect sizes were determined with the r statistic, calculated by dividing the absolute Z value by the square root of number of pairs. All statistical analyses were performed with SPSS v30 (IBM Corp). Given the exploratory nature of the investigation, two-sided tests were used with an unadjusted significance level of p ≤ 0.05.

## Results

### Descriptive Characteristics

Sample characteristics are displayed in Table 2. About half of patients were diagnosed with low-grade tumors (53% WHO grade II) and all lesions were near left hemisphere language-eloquent regions. Most patients (~90%) were maintained on antiepileptic medications at both pre- and postoperative imaging sessions and around half the sample was on steroids at both timepoints. Stereotactic biopsy prior to surgical tumor resection was performed in 67% of patients. All patients underwent awake craniotomy with language mapping and 74% had gross or near total resection per T1-weighted MRI [% Resected M(SD) = 92.7 (15.8)] and 70% with gross or near total resection per FLAIR MRI [% Resected M(SD) = 85.2 (15.4)]. Restricted diffusion on DWI in the immediate postoperative setting (24 to 48 h) was identified in 53% of patients, but area of restriction was considered small or minimal in most (75%). Surgical technique for all patients included direct electrical stimulation language mapping and image-guided (MRI and/or ultrasound) microsurgical resection. Although an object naming paradigm was utilized for mapping in all patients, protocols varied on a case-by-case basis according to brain regions of interest and unique patient needs/concerns. On average, neuropsychological assessment was completed about 3 days prior to [M(SD) = 2.8 (2.1) days] and 4 weeks following resection [M(SD) = 33.2 (27.1) days], and functional imaging was performed about 2 days prior to [M(SD) = 2.3 (2.2) days] and between 2 and 3 weeks after surgery [M(SD) = 17.1 (6.4) days].

**Table 2.** Demographic and clinical characteristics

	N = 15
Age, years M (SD)	44.6 (14.9)
% Female	53
% White	87
% Right Hand Dominant	100
Education, years M (SD)	16.0 (2.7)
Seizure History, % yes	80
AED at Preop Scan, % yes	87
AED at Postop Scan, % yes	93
Steroid at Preop Scan, % yes	53
Steroid at Postop Scan, % yes	53
Preop Scan to Resection, days M (SD)	2.3 (2.2)
Resection to Postop Scan, days M (SD)	17.1 (6.4)
Preop Cognitive Testing to Resection, days M (SD)	2.8 (2.1)
Resection to Postop Cognitive Testing, days M (SD)	33.2 (27.1)
Hemisphere Lesion, % Left	100
Lesion Location, %	
Frontal	47
Temporal	40
Parietal	13
FLAIR Volume	
Preop, cm^3^ M (SD)	61.4 (37.1)
Postop, cm^3^ M (SD)	10.7 (13.6)
Residual, % M (SD)	15.9 (15.4)
T1-Weighted Volume^a^	
Preop, cm^3^ M (SD)	37.6 (26.5)
Postop, cm^3^ M (SD)	2.7 (6.1)
Residual, % M (SD)	7.3 (15.8)
FLAIR Extent of Resection, %	
Gross Total	47
Near Total	20
Subtotal	33
T1 Extent of Resection,^a^ %	
Gross Total	67
Near Total	7
Subtotal	26
Awake Craniotomy, % yes	100
Prior Intervention, %	
None	33
Biopsy	67
Tumor Grade, %	
II	53
III	7
IV	40
Histology, %	
Glioblastoma	40
Astrocytoma	47
Oligodendroglioma	13

Abbreviation: AED, antiepileptic drug.

^a^Greatest of T1-weighted contrast-enhancing (hyperintense) or non-enhancing (hypointense) volume.

### Functional Independence Outcomes

**Figure 1.**
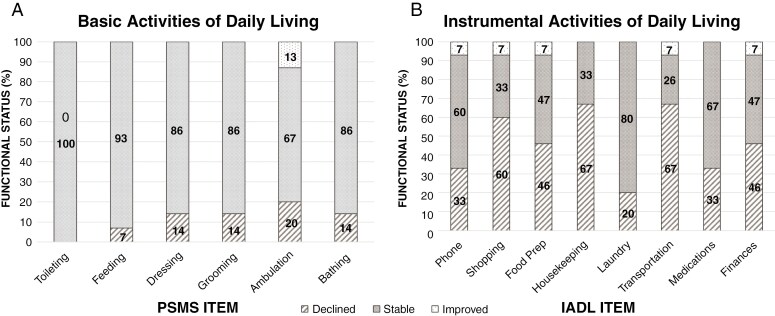
Rates of postoperative change in instrumental and basic activities of daily living.

Pre- and postoperative Total IADL and PSMS scores are displayed in Table 3. Pre-operatively, 80% of patients required no assistance in PSMS items, and overall independence did not significantly change postoperatively at the group level (PSMS Total Mdn change = 0.0). At the item-level, need for assistance on at least 1 PSMS item increased in 27% of patients, with reduced independence observed in less than 15% of patients across all PSMS items (see Figure 1A). In contrast, 53% of the sample exhibited need for assistance on at least 1 IADL item preoperatively. Postoperatively, overall need for assistance on the IADL significantly increased (Mdn change = 4.0, *P* = .006), with a medium to large effect size. At the item-level, 80% of patients declined in functional independence on at least 1 IADL item, most frequently in Transportation, Shopping, and Housekeeping (see Figure 1B). Given State regulations upon driving related to seizure history, sensitivity analyses were conducted to examine whether increased postoperative need for assistance with Transportation was related to postoperative change in seizure status. Importantly, 80% of this newly diagnosed sample had positive seizure history at baseline evaluation, which was unchanged at follow-up about 1 month later, indicating that alterations in independence in this domain were unlikely to be attributable to seizure restriction.

As indicated in Table 3, pre- and postoperative KPS scores did not significantly differ at the group level (KPS Mdn change = 0.0). While about 35% of patients (*N* = 5) exhibited a postoperative KPS decline of 10 points, only a single patient showed worsening of at least 20 points. Postoperative KPS remained 80 or greater in all but one patient.

### Motor and Neurocognitive Function, Clinical Characteristics, and Functional Independence

No postoperative gross motor worsening was noted on neurologic exam. At the group level, Grooved Pegboard performances did not significantly change postoperatively for either the right or left hand (see Table 3). Using RCI criteria, no patients exhibited significant postoperative decline on Grooved Pegboard for the dominant right hand, and only a single patient declined with the left hand. Pre- to postoperative change (ie, difference scores) in PSMS, IADL, and KPS were not associated with motor outcomes, antiepileptic or steroid use, tumor volume, extent of resection, or presence of restricted diffusion. Tumor grade was not associated with pre- to postoperative change for KPS or PSMS and IADL Total scores/items, with the exception of Food Preparation on the IADL. Increased postoperative need for assistance in Food Preparation was observed in patients with high-grade tumors (Mdn change = 1.0, IQR = 3.0) compared to those with low-grade lesions (Mdn change = 0.0, IQR = 0.75; Z = −2.10, *P* = .036). Change scores on PSMS and IADL measures were not associated with change in KPS ratings, whether as a continuous measure or classified binarily from 10- or 20-point change criteria. Change scores on independence measures were also not associated with primary lobe involved (frontal vs. temporal), age, gender, or education.

As previously reported^16^ and summarized in Table 3, neurocognitive functioning significantly declined postoperatively in aspects of verbal memory (HVLT-R Recognition: Z = −2.11, *P* = .035), processing speed (TMT Part A: Z = −2.33, *P* = .020), executive functioning (TMT Part B: Z = −2.33, *P* = .020; Similarities: Z = −2.48, *P* = .013; COWA: Z = −2.48, *P* = .013), and language comprehension (Token; Z = −2.67, *P* = .008). Rates of RCI decline were most frequent in the same domains, including verbal memory (HVLT-R: Total Recall, 53%; Delayed Recall, 53%; Recognition, 46%), aspects of executive functioning (TMT Part B, 54%; Similarities, 46%; COWA, 33%), and language (Token Test, 35%).

**Figure 2.**
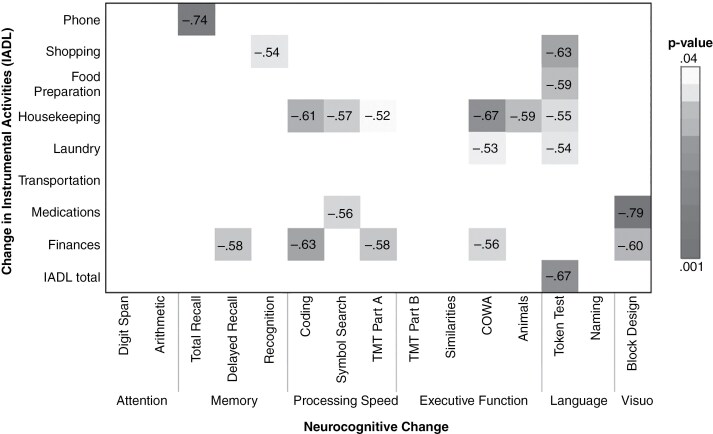
Significant associations between pre- to postoperative changes in ratings of instrumental activities of daily living and neurocognitive performances. Note. Values represent correlation coefficients (ρ).

Significant associations between pre- to postoperative change (ie, difference) scores in neurocognitive functioning and IADL ratings are displayed in Figure 2. Medium to large inverse relationships (ρ = −0.52 to −0.79, all *P* < .05) were observed between changes in neurocognitive performance and daily functional abilities, with decline in multiple neurocognitive domains associated with reduced independence in IADL tasks. Housekeeping and Financial Management appeared associated with the greatest number of neurocognitive tests (5 and 6, respectively), and the strongest associations (∣ρ∣ > 0.65) were noted between learning aspects of memory (Total Recall) and Phone Use, verbal fluency aspects of executive functioning (COWA) and Housekeeping, and comprehension aspects of language (Token Test) and Total scores. Interestingly, worsening in visuospatial functioning (Block Design) was associated with reduced ability to manage Finances and Medications. Correlational analyses between changes on neurocognitive tests and basic activities of daily living were not possible due to general stability across pre- to postoperative PSMS ratings.

### Functional Connectomics

Compared to preoperative levels, significant postoperative reductions with medium to large effect sizes were found in Betweenness Centrality and Assortativity (see Table 2). Pre- to postoperative changes (ie, difference scores) in connectomic properties were not significantly associated with tumor grade, pre- or postoperative lesion volume, extent of resection, presence of restricted diffusion, or antiepileptic and steroid medication use.

#### Associations between changes in connectomics and patient outcomes

Pre- to postoperative PSMS Total change scores were not significantly associated with changes in any connectomic properties. However, pre- to postoperative change in Clustering Coefficient was positively associated with change in need for assistance in Bathing [ρ(13) = 0.59, *P* = .021] and Grooming [ρ(13) = 0.59, *P* = .021] on the PSMS. Of note, patients with increased postoperative need for assistance in Bathing represented the same as those requiring greater assistance in Grooming, explaining the identical associations with change in Clustering Coefficient.

**Figure 3.**
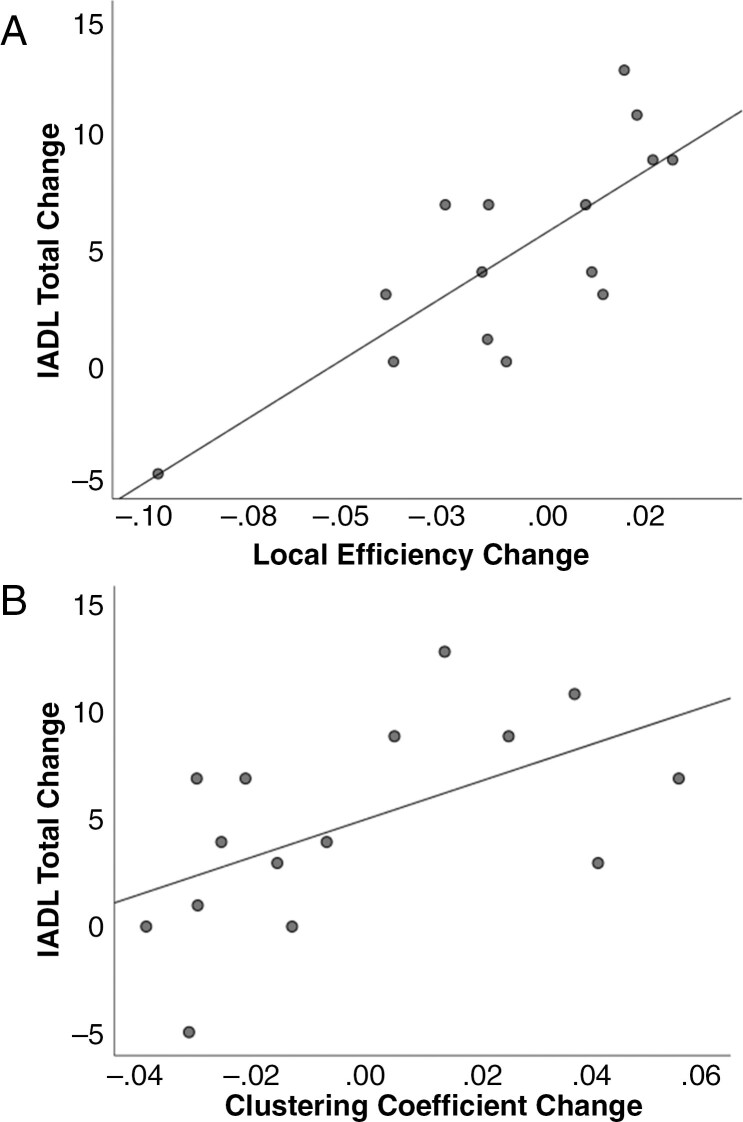
Scatterplot of the relationship between alterations in connectomic properties and changes in overall independence in instrumental activities. A, Change in Instrumental Activities of Daily Living scale (IADL) Total scores plotted against alterations in local efficiency. B, Change in IADL Total scores plotted against alterations in clustering coefficient.

Pre- to postoperative IADL Total change scores were positively associated with changes in Local Efficiency [ρ(13) = 0.72, *P* = .002] and Clustering Coefficient [ρ(13) = .59, *P* = 0.022] (see Figure 3). At the IADL item level, pre- to postoperative change in Local Efficiency was positively associated with need for assistance in Shopping [ρ(13) = 0.71, *P* = .003], Food Preparation [ρ(13) = 0.61, *P* = .019], and Financial Management [ρ(13) = 0.58, *P* = .022]. Change in Clustering Coefficient was positively associated with need for assistance in Telephone Use [ρ(13) = 0.56, *P* = .030] and Housekeeping [ρ(13) = 0.52, *P* = .047]. Change in need for assistance with Telephone Use was also positively associated with change in Betweenness Centrality [ρ(13) = 0.58, *P* = .025] and Path Length [ρ(13) = 0.55, *P* = .033].

In summary of prior results,^16^ large magnitude associations were evident between pre- to postoperative changes in connectomic properties and neurocognitive functioning, including auditory comprehension (Token Test) and Clustering Coefficient [ρ(12) = −0.55, *P* = .043], executive functioning (COWA) and Assortativity [ρ(13) = −0.53, *P* = .041], as well as verbal memory (HVLT-R) and Betweenness Centrality [Total Recall: ρ(12) = −0.78, *P* = .001; Delayed Reall: ρ(13) = −0.60, *P* = .017], Assortativity [Delayed Recall: ρ(13) = −0.58, *P* = .023], and Local Efficiency [Recognition: ρ(13) = −0.54, *P* = .036].

Given time lag between postoperative fMRI and neuropsychological evaluation, sensitivity analyses were conducted to investigate whether variation in this gap was associated with pre- to postoperative connectomic, functional independence, or neurocognitive changes, or influenced the associations between connectomics and outcomes. Neurocognitive testing lagged fMRI an average of 2 weeks [M(SD) = 16.1 (14.5) days], though time interval was not significantly associated with changes in connectomics or any patient outcomes. Additionally, associations between outcomes and connectomic changes remained significant when covarying for lag in partial correlational analyses.

## Discussion

Consideration of potential treatment impact upon daily functioning is increasingly recognized as critical for patients with glioma,^6^ particularly given that acquired decline can have profound impact upon patient quality of life during an often-limited survival period. This study provides preliminary evidence that postoperative decline in functional independence persists into the early recovery period and is common following resection of lesions near language-eloquent cortex, particularly for more complex daily activities. At around 1 month postoperatively, overall informant-rated independence in instrumental activities was reduced compared to preoperative levels, with the majority of the sample exhibiting more need for assistance in at least 1 daily function. Specific activities most affected included shopping, housekeeping, and transportation, with 60–67% of the sample showing postoperative worsening. The frequent early postoperative loss of independence in instrumental activities contrasts with the relatively stable rates of capability to perform basic activities of daily living. Physician-rated functional status was also generally unchanged postoperatively, consistent with prior research in similar brain tumor populations.^6,7,46^

The more common decline noted in instrumental activities underscores the importance of using measures that assess a broad range of functions important to patient independence and associated quality of life. The discrepancy in informant-reported independence in instrumental activities and physician-rated KPS scores suggests the IADL may be more sensitive to changes in complex higher-order daily functions. It could also be the case that caregiver-informants are better able to identify patient limitations in daily activities given opportunity to observe patient capabilities over longer periods of time in diverse real-world settings. Unfortunately, there is a paucity of data on pre- to postoperative outcomes on dedicated measures of activities of daily living in this population.^6^ As such, it is difficult to determine the generalizability of our findings, especially given the modest sample size. Nonetheless, we expect our rates of decline in independence are reflective, at least in part, of the relatively early postoperative assessment timepoint in conjunction with the high-risk lesion location.

Prevention of acquired decline in functional independence, even if largely transient in some patients, remains an important goal given the impact upon quality of life in the context of an often-limited survival duration and high caregiver burden. In our sample, postoperative changes in performance status and functional independence were not associated with tumoral features, aside from higher tumor grade relating to increased need for assistance in a single instrumental activity (food preparation). Accordingly, clinical structural imaging alone is likely not sufficient for informing risk of postoperative decline in daily activities. In contrast, the strong relationships between pre- to early postoperative alterations in functional connectomic properties and patient independence suggest that brain connectivity metrics may eventually become a useful tool for outcome prediction and surgical planning.

To our knowledge, this study represents the first to assess connectomic features associated with functional independence following glioma resection. Interestingly, connectomic features with the strongest and most consistent associations with functional outcomes comprised measures of network segregation (clustering coefficient and local efficiency). More specifically, clustering coefficient represents the extent to which nodes in a network are interconnected, somewhat analogous to whether a group of individuals comprises a more or less tightly knit social “clique.” Local efficiency reflects a similar construct, but is measured by considering communication between neighbors when a member (ie, node) is removed. Those showing postoperative increases in these metrics exhibited greatest increase in assistance needed across multiple domains, including complex tasks involving telephone use, housekeeping, shopping, and finances, as well as a couple of more basic (and interrelated) self-care activities (bathing and grooming). Conceptually, this suggests that as groups of neighboring nodes become increasingly locally interconnected following surgery, resulting in tight-knit “cliques,” whole-brain communication and integration may be compromised making complex activities more difficult to execute independently. It should be noted that patients in this study did not acquire gross motor deficits or decline in manual dexterity that could explain the postoperative worsening in daily activities. Further, increase in postoperative need for assistance with transportation was not explained by seizure status. Accordingly, these preliminary findings suggest that surgical impact upon independence may relate more to disruption of networks underlying the neurobehavioral capacity to perform various daily activities rather than fundamental motor skills or regulatory restrictions (eg, driving laws).

Our previous work demonstrated that neurocognitive impairment, particularly verbal learning and executive function deficits, predict functional independence in patients with glioma.^2^ Consistent with this, we identified strong relationships between pre- to postoperative changes in neurocognition and independence in instrumental activities in this sample of patients with eloquent glioma. Intuitively, these relationships might suggest shared underlying neural mechanisms across these patient outcomes, such as alterations in aspects of functional connectivity. However, the connectomic features associated with daily functional decline in this study appear somewhat different from those most related to neurocognitive decline, with the latter primarily comprised of measures reflecting network centrality or “hubness.”^16^ This may relate to the fact that patient independence not only relies upon isolated neurocognitive functions, but requires the coordinated integration of these various neurocognitive domains. This contention is supported by the observation that specific instrumental tasks (eg, finances, housekeeping) are strongly associated with multiple neurocognitive domains (eg, memory, speed, executive functioning), underscoring the complex integration of cognitive abilities needed to successfully execute instrumental daily activities. This is also consistent with the finding that increased segregation conveys poorer outcome regarding daily independence.

Optimal brain networks involve a small-world architecture, balancing high local clustering and short average path length, allowing for efficient processing and information transfer.^47^ Disruption of these features can occur in a bidirectional fashion, including breakdown of or pathological increase in small-worldness. It is known that gliomas have wide ranging effects on network integrity, impacting connectivity both locally and globally.^48^ Surgery can further perturb functional connectivity in a pathological fashion, including maladaptive hyper- and hypoconnectivity.^23,24^ However, it should be noted that postoperative changes in functional network activity can also have a salutatory impact. Indeed, our prior work identified some patients that exhibited stable or even improved neurocognitive functioning postoperatively,^16^ and more positive outcome was associated with reductions in connectivity metrics. Accordingly, in contrast to network disruption in some patients, surgery might reduce pathological tumor-related hyperconnectivity in others, as has been observed in prior studies.^49,50^ Understanding how to modulate these changes through surgical planning represents a critical next step in harnessing functional connectomics to improve patient outcomes.

### Future Directions and Limitations

Restriction of our sample to patients with language-eloquent tumors was deliberate given particular risk of postoperartive neurocognitive and daily functional decline related to resection involving these areas. However, we acknowledge examination of connectomic changes might also inform variation in outcomes across patients with glioma of any brain region. Future work incorporating a more heterogeneous tumor distribution would be helpful to better understand regional contributors to connectomic changes, associated functional declines, and recovery trajectories. Despite language-eloquent tumor location, most patients in our sample are expected to improve with additional time for recovery (barring disease progression and/or adverse impact of adjuvant treatment). It is also known that some will not return to their baseline level of functioning and independence.^18^ Studies with further longitudinal follow-up remain needed to better appreciate recovery curves and identify risk factors associated with persisting functional decline. Identification of predictors of poorer outcomes in functional independence, both early and later in the postoperative period, may enable development of better strategies to mitigate postoperative decline.

The relatively small sample size represents a primary limitation of this work, and findings must be contextualized within the specific clinical characteristics of this cohort, including mixed histopathological diagnoses and most patients presenting with positive seizure history and antiepileptic and/or steroid use, which could impact connectomic properties and patient outcomes. Additionally, sample size and variation in brain mapping protocols across cases prevented investigation of relationships between connectomic and independence changes with intraoperative brain mapping findings. Future work with well-characterized and more standardized mapping protocols could elucidate how intraoperative data might contribute to network and functional changes. It is also important to note that postoperative neurocognitive testing lagged fMRI by an average of 2 weeks, and tighter coordination of timing between neuropsychological assessment and neuroimaging would be ideal. Nonetheless, within this context findings suggest that early postoperative changes in the functional connectome may be useful in prognosticating patient outcomes, even regarding informant-rated functional abilities occurring a few weeks after imaging is acquired. Finally, while use of caregiver informants for rating need for assistance reduces risk of bias associated with patient neurocognitive impairment, reduced insight, and affective distress, use of more “ecologically-valid” performance-based measures would be ideal. Future work incorporating such measures alongside informant ratings would be clarifying, especially for determining rates of objective decline in daily activity abilities.

Despite these limitations, very few studies have acquired resting-state fMRI both pre- and postoperatively in this rare disease population, with most investigations limited by similarly modest sample sizes and heterogeneity. Further, none to our knowledge have incorporated longitudinal functional imaging data with functional independence outcomes, and the strong associations noted are not only hypothesis-generating but also encouraging for the future development of connectomics-based outcome prediction models in larger and more heterogeneous samples. Larger-scale investigations would enable consideration of additional potential contributors to postoperative connectomic changes and patient outcomes. Potentially important variables include specific surgical techniques utilized, intraoperative mapping results, histologic and molecular tumor subtypes, seizure activity and medication effects, impact of perioperative infarct volume, differences according to precise lesion localization, and social determinants of brain health. Structural equation modeling approaches could investigate the interplay between changes in network properties, neurocognition, and daily activities, clarifying these relationships and elucidating potential causal and/or mediating pathways. Consideration of impact of different imaging processing and analytic approaches would also be informative, including use of more advanced connectivity thresholding methods such as orthogonal minimum spanning tree. Finally, incorporating longer-term follow-up is needed to better clarify how well early postoperative connectomic changes predict patient recovery trajectories over time. This would also allow understanding of how early connectomic changes might predict other important outcomes, such as return to work and disability status, which represent critical contributors to patient quality of life.

Implications of findings upon clinical decision making cannot be concluded from the present study, as resting-state fMRI and derived network properties were not considered in surgical planning. Although connectomics hold promise to eventually serve this purpose, it is essential to first determine that surgery-related network perturbations reliable influence outcomes, supporting the plausibility that mitigation of these changes can improve outcomes. Future larger scale work should explore how resection or sparing of specific preoperative network features influence postoperative alterations in the connectome. Ultimately, this may lead to surgical planning approaches that reduce risk to critical whole-brain network properties and better preserve patient function. By selecting approaches associated with less connectomic disruption and functional decline, the early postoperative functioning “set point” for rehabilitation and recovery may be improved. Such hastening of the recovery trajectory represents a particularly salient goal in conditions with poor prognosis such as malignant glioma.

## Conclusions

This work lays the foundation for understanding whole-brain connectomic changes associated with early postoperative daily functional decline in patients with language-eloquent glioma. With a better understanding of these relationships, future research may enable better prediction of network changes and improve surgical planning, postoperative outcomes, and enhance recovery trajectories.

## Data Availability

Data will be made available by reasonable request to the corresponding author.

## Notes

**Previous Presentation** This work was previously presented as an oral report at the 28^th^ annual scientific meeting of the Society for Neuro-Oncology, Vancouver, BC, Canada, on November 18, 2023. This work represents a novel extension of previously published data which are summarized herein. [Neurosurgery 2021;88(3):544-551; doi:10.1093/neuros/nyaa453]

## References

[1] Taphoorn MJB , Klein M. Cognitive deficits in adult patients with brain tumours. Lancet Neurol. 2004;3(3):159–168. 14980531 10.1016/S1474-4422(04)00680-5

[2] Noll KR , Bradshaw ME, Weinberg JS, Wefel JS. Neurocognitive functioning is associated with functional independence in newly diagnosed patients with temporal lobe glioma. Neurooncol. Pract.. 2018;5(3):184–193. 30094046 10.1093/nop/npx028 PMC6075221

[3] Sanai N , Polley M-Y, McDermott MW, Parsa AT, Berger MS. An extent of resection threshold for newly diagnosed glioblastomas. J Neurosurg. 2011;115(1):3–8. 21417701 10.3171/2011.2.jns10998

[4] Li YM , Suki D, Hess K, Sawaya R. The influence of maximum safe resection of glioblastoma on survival in 1229 patients: can we do better than gross-total resection? J Neurosurg. 2016;124(4):977–988. 26495941 10.3171/2015.5.JNS142087

[5] Lacroix M , Abi-Said D, Fourney DR, et al A multivariate analysis of 416 patients with glioblastoma multiforme: prognosis, extent of resection, and survival. J Neurosurg. 2001;95(2):190–198. 10.3171/jns.2001.95.2.0190 11780887

[6] Hamer PC , Klein M, Hervey-Jumper SL, Wefel JS, Berger MS. Functional outcomes and health-related quality of life following glioma surgery. Neurosurg. 2021; 88(4):720–732. 10.1093/neuros/nyaa365 PMC7955971 33517431

[7] Chaichana KL , Cabrera-Aldana EE, Jusue-Torres I, et al When gross total resection of a glioblastoma is possible, how much resection should be achieved? World Neurosurg. 2014;82(1-2):e257–e265. 24508595 10.1016/j.wneu.2014.01.019

[8] Bonifazi S , Passamonti C, Vecchioni S, et al Cognitive and linguistic outcomes after awake craniotomy in patients with high-grade gliomas. Clin Neurol Neurosurg. 2020;198:106089. 32738586 10.1016/j.clineuro.2020.106089

[9] Sanai N , Mirzadeh Z, Berger MS. Functional outcome after language mapping for glioma resection. N Eng J Med. 2008;358(1):18–27. 10.1056/NEJMoa067819 18172171

[10] Bello L , Gambini A, Castellano A, et al Motor and language DTI Fiber Tracking combined with intraoperative subcortical mapping for surgical removal of gliomas. Neuroimage. 2008;39(1):369–382. 17911032 10.1016/j.neuroimage.2007.08.031

[11] Duffau H , Capelle L, Sichez N, et al Intraoperative mapping of the subcortical language pathways using direct stimulations: an anatomo‐functional study. Brain. 2002;125(1):199–214. 11834604 10.1093/brain/awf016

[12] Kamada K , Todo T, Masutani Y, et al Combined use of tractography-integrated functional neuronavigation and direct fiber stimulation. J Neurosurg. 2005;102(4):664–672. 15871509 10.3171/jns.2005.102.4.0664

[13] Hendi K , Rahmani M, Larijani A, et al Changes in cognitive functioning after surgical resection of language-related, eloquent-area, high-grade gliomas under awake craniotomy. Cogn Behav Neurol. 2022;35(2):130–139. 35486526 10.1097/WNN.0000000000000307

[14] Bu LH , Zhang J, Lu JF, Wu JS. Glioma surgery with awake language mapping versus generalized anesthesia: a systematic review. Neurosurg Rev. 2021;44(4):1997–2011. 33089447 10.1007/s10143-020-01418-9

[15] Noll KR , Weinberg JS, Ziu M, et al Neurocognitive changes associated with surgical resection of left and right temporal lobe glioma. Neurosurg. 2015;77(5):777–785. 10.1227/NEU.0000000000000987 PMC4831270 26317672

[16] Noll KR , Chen HS, Wefel JS, et al Alterations in functional connectomics associated with neurocognitive changes following glioma resection. Neurosurg. 2021;88(3):544–551. 10.1093/neuros/nyaa453 PMC7884148 33080024

[17] Habets EJ , Kloet A, Walchenbach R, et al Tumour and surgery effects on cognitive functioning in high-grade glioma patients. Acta Neurochir. 2014;156(8):1451–1459. 24879620 10.1007/s00701-014-2115-8

[18] Satoer D , Visch-Brink E, Dirven C, Vincent A. Glioma surgery in eloquent areas: can we preserve cognition? Acta Neurochir. 2016;158(1):35–50. 26566782 10.1007/s00701-015-2601-7 PMC4684586

[19] Yuan B , Zhang N, Gong F, et al Longitudinal assessment of network reorganizations and language recovery in postoperative patients with glioma. Brain Commun. 2022;4(2):fcac046. 35415604 10.1093/braincomms/fcac046 PMC8994117

[20] Lakhani DA , Sabsevitz DS, Chaichana KL, Quiñones-Hinojosa A, Middlebrooks EH. Current state of functional MRI in the presurgical planning of brain tumors. Radiol Imaging Cancer. 2023;5(6):e230078. 37861422 10.1148/rycan.230078 PMC10698604

[21] Spronk M , Keane BP, Ito T, et al A whole-brain and cross-diagnostic perspective on functional brain network dysfunction. Cereb Cortex. 2021;31(1):547–561. 32909037 10.1093/cercor/bhaa242 PMC7947178

[22] Sporns O. Structure and function of complex brain networks. Dialogues Clin Neurosci. 2013;15(3):247–262. 24174898 10.31887/DCNS.2013.15.3/osporns PMC3811098

[23] Carbo EW , Hillebrand A, Van Dellen E, et al Dynamic hub load predicts cognitive decline after resective neurosurgery. Sci Rep. 2017;7(1):42117. 28169349 10.1038/srep42117 PMC5294457

[24] Sparacia G , Parla G, Re VL, et al Resting-state functional connectome in patients with brain tumors before and after surgical resection. World Neurosurg. 2020;141:e182–e194. 32428723 10.1016/j.wneu.2020.05.054

[25] Agcaoglu O , Miller R, Mayer AR, Hugdahl K, Calhoun VD. Lateralization of resting state networks and relationship to age and gender. Neuroimage. 2015;104:310–325. 25241084 10.1016/j.neuroimage.2014.09.001 PMC4252729

[26] Pertz M , Kowalski T, Jetschke K, et al Pre-and postoperative self-reported and objectively assessed neurocognitive functioning in lower grade glioma patients. J Clin Neurosci. 2022;106:185–193. 36369078 10.1016/j.jocn.2022.10.026

[27] Sawaya R , Hammoud M, Schoppa D, et al Neurosurgical outcomes in a modern series of 400 craniotomies for treatment of parenchymal tumors. Neurosurg. 1998;42(5):1044–1055. 10.1097/00006123-199805000-00054 9588549

[28] Lawton MP , Brody EM. Assessment of older people: self-maintaining and instrumental activities of daily living. Gerontologist. 1969;9(3):179–186. 5349366

[29] Edwards MM. The reliability and validity of self-report activities of daily living scales. Can J Occup Ther. 1990;57(5):273–278.

[30] Sikkes SA , De Lange-de Klerk ES, Pijnenburg YA, Scheltens P, Uitdehaag BMJ. A systematic review of Instrumental Activities of Daily Living scales in dementia: room for improvement. J Neurol Neurosurg Psychiatry. 2009;80(1):7–12. 19091706 10.1136/jnnp.2008.155838

[31] Oort Q , Taphoorn MJ, Sikkes SA, et al Evaluation of the content coverage of questionnaires containing basic and instrumental activities of daily living (ADL) used in adult patients with brain tumors. J Neurooncol. 2019;143(1):1–3. 30887244 10.1007/s11060-019-03136-9 PMC6482128

[32] Chambless LB , Kistka HM, Parker SL, et al The relative value of postoperative versus preoperative Karnofsky Performance Scale scores as a predictor of survival after surgical resection of glioblastoma multiforme. J Neurooncol. 2015;121(2):359–364. 25344883 10.1007/s11060-014-1640-x

[33] Sherman E , Hrabok M. A Compendium of Neuropsychological Tests: Fundamentals of Neuropsychological Assessment and Test Reviews for Clinical Practice. Oxford University Press; 2023.

[34] Noll KR , Ziu M, Weinberg JS, Wefel JS. Neurocognitive functioning in patients with glioma of the left and right temporal lobes. J Neurooncol. 2016;128(2):323–331. 27022915 10.1007/s11060-016-2114-0 PMC4884162

[35] Heaton RK. Revised Comprehensive Norms for an Expanded Halstead-Reitan Battery: Demographically Adjusted Neuropsychological Norms for African American and Caucasian Adults, Professional Manual. Psychological Assessment Resources; 2004.

[36] Chelune GJ , Naugle RI, Lüders H, Sedlak J, Awad IA. Individual change after epilepsy surgery: practice effects and base-rate information. Neuropsychology. 1993;7(1):41–52.

[37] Kumar VA , Lee J, Liu HL, et al Recommended resting-state fMRI acquisition and preprocessing steps for preoperative mapping of language and motor and visual areas in adult and pediatric patients with brain tumors and epilepsy. American Journal of Neuroradiology. 2024;45(2):139–148. 38164572 10.3174/ajnr.A8067 PMC11285996

[38] Whitfield-Gabrieli S , Nieto-Castanon AC. a functional connectivity toolbox for correlated and anticorrelated brain networks. Brain Connect. 2012;2(3):125–141. 22642651 10.1089/brain.2012.0073

[39] Rachakonda S , Egolf E, Correa N, Calhoun V. Group ICA of fMRI toolbox (GIFT) manual. http://www.nitrc.org/docman/view.php/55/295/v1.3d. Accessed November 1, 2023.

[40] Tzourio-Mazoyer N , Landeau B, Papathanassiou D, et al Automated anatomical labeling of activations in SPM using a macroscopic anatomical parcellation of the MNI MRI single-subject brain. Neuroimage. 2002;15(1):273–289. 11771995 10.1006/nimg.2001.0978

[41] Ashburner JA. fast diffeomorphic image registration algorithm. Neuroimage. 2007;38(1):95–113. 17761438 10.1016/j.neuroimage.2007.07.007

[42] Luppi AI , Gellersen HM, Liu ZQ, et al Systematic evaluation of fMRI data-processing pipelines for consistent functional connectomics. Nat Commun. 2024;15(1):4745. 38834553 10.1038/s41467-024-48781-5 PMC11150439

[43] Rubinov M , Sporns O. Complex network measures of brain connectivity: uses and interpretations. Neuroimage. 2010;52(3):1059–1069. 19819337 10.1016/j.neuroimage.2009.10.003

[44] Welton T , Kent DA, Auer DP, Dineen RA. Reproducibility of graph-theoretic brain network metrics: a systematic review. Brain Connect. 2015;5(4):193–202. 25490902 10.1089/brain.2014.0313 PMC4432917

[45] Cohen J. Statistical Power Analysis for the Behavioral Sciences. 2nd ed. Lawrence Earlbaum Associates; 1988.

[46] Chaichana KL , Halthore AN, Parker SL, et al Factors involved in maintaining prolonged functional independence following supratentorial glioblastoma resection. J Neurosurg. 2011;114(3):604–612. 20524825 10.3171/2010.4.JNS091340 PMC3725949

[47] Wang R , Liu M, Cheng X, et al Segregation, integration, and balance of large-scale resting brain networks configure different cognitive abilities. Proc Natl Acad Sci USA. 2021;118(23):e2022288118. 34074762 10.1073/pnas.2022288118 PMC8201916

[48] Nenning KH , Furtner J, Kiesel B, et al Distributed changes of the functional connectome in patients with glioblastoma. Sci Rep. 2020;10(1):18312. 33110138 10.1038/s41598-020-74726-1 PMC7591862

[49] Zimmermann ML , Breedt LC, Centeno EG, et al The relationship between pathological brain activity and functional network connectivity in glioma patients. J Neurooncol. 2024;3(3):1–1. 10.1007/s11060-024-04577-7 PMC10876827 38308803

[50] Douw L , Baayen H, Bosma I, et al Treatment-related changes in functional connectivity in brain tumor patients: a magnetoencephalography study. Exp Neurol. 2008;212(2):285–290. 18534578 10.1016/j.expneurol.2008.03.013

